# Unraveling the Potential Antiviral Activity of Isoxazoline-Carbocyclic Monophosphate Nucleotides Against Echovirus 11

**DOI:** 10.3390/microorganisms13122662

**Published:** 2025-11-23

**Authors:** Emilia Palazzotto, Valeria Stefanizzi, Floriana Bonura, Federica Cacioppo, Marco Leusciatti, Paolo Quadrelli, Annalisa Chianese, Carla Zannella, Anna De Filippis, Antonio Mastino, Francesca Marino Merlo, Simona De Grazia

**Affiliations:** 1 Department of Health Promotion, Mother and Child Care, Internal Medicine and Medical Specialties “G. D’Alessandro”, University of Palermo, Viale del Vespro Palermo 133, 90127 Palermo, Italy; floriana.bonura@unipa.it (F.B.); federica-cacioppo@libero.it (F.C.); simona.degrazia@unipa.it (S.D.G.); 2 Department of Chemical, Biological, Pharmaceutical, and Environmental Sciences, University of Messina, Viale Ferdinando Stagno d’Alcontres 31, 98166 Messina, Italy; valeria.stefanizzi@unime.it (V.S.); antonio.mastino@ift.cnr.it (A.M.); francesca.marino@unime.it (F.M.M.); 3 INSTM Research Unit of Pavia, Department of Chemistry, University of Pavia, Viale Taramelli 10-12, 27100 Pavia, Italy; marco.leusciatti01@universitadipavia.it (M.L.); paolo.quadrelli@unipv.it (P.Q.); 4 Department of Experimental Medicine, University of Campania “Luigi Vanvitelli”, 80138 Naples, Italy; annalisa.chianese@unicampania.it (A.C.); carla.zannella@unicampania.it (C.Z.); anna.defilippis@unicampania.it (A.D.F.); 5 The Institute of Translational Pharmacology, CNR, Via Fosso del Cavaliere 100, 00133 Roma, Italy

**Keywords:** Echovirus 11, enterovirus, antiviral activity, isoxazoline-carbocyclic monophosphate nucleotides

## Abstract

From July 2022, a novel Echovirus 11 (E11) variant has been associated with severe neonatal infections and liver failure. Currently, there are no vaccines or antiviral options for the targeted treatment of non-polio enterovirus (EV) infections; therefore, anti-EV drugs are urgently needed. In this study, the putative anti-E11 activity of two isoxzoline-carbocyclic monophosphate nucleotides (**4a** and **4b**) was evaluated in vitro by cytopathic effect (CPE) reduction in VERO 76 cells and qRT-PCR. Treatment with nucleotide **4a** at 25 and 50 μM successfully diminished the CPE caused by E11 by 90% and 75%, respectively, and induced a reduction in viral RNA in the supernatant by 72% and 89%. In contrast, the treatment with 25 and 50 μM of **4b** caused a minor inhibition of CPE (58 and 38%), and no significant E11 RNA level changes were observed. A time course viral progeny production assay was performed to assess the inhibitory effect of nucleotide **4a** on E11 infection progression. Compared to the control, the treated group showed a significant drop in viral RNA levels, with reductions of 43% at 10 h, 65% at 24 h, and 96% at 48 h post-infection. The results showed the extensive antiviral properties of the monophosphate nucleotide **4a** in vitro. Moreover, a retrospective molecular docking study strongly supports that nucleotide **4a** is an RdRp inhibitor capable of decreasing E11 genome replication and virus particle formation in VERO 76 cells.

## 1. Introduction

Emerging and reemerging viruses represent a constant epidemic threat not only for human and animal health, but also due to the economic, social, and political effects they may produce globally. Climate change, massive international travel, fast urbanization, increased population density, and proximity to wild animals contribute to the increased number of viral emergencies [1]. The recent SARS-CoV-2 outbreak dramatically highlighted how unprepared we are to respond to such events and how limited our therapeutic arsenal is against them.

Echoviruses are small (~300 Å in diameter—27 nm), non-enveloped icosahedral viruses with a single-stranded RNA genome that belong to the *Enterovirus B* species of the family *Picornaviridae,* with at least 28 serotypes [2]. The primary modes of transmission for enteroviruses (EV) are through the fecal-oral route and respiratory pathways, although instances of vertical and postnatal transmission, particularly in hospital environments, have also been documented [3]. Infections caused by non-polio EV can lead to several health issues, ranging from mild, nonspecific inflammatory responses to conditions like skin rashes, fevers, diarrhea, herpangina, and hand, foot, and mouth disease (HFMD), or severe respiratory infection and polio-like paralytic illnesses [4]. Specific echovirus serotypes can cause severe illness in neonates or other vulnerable populations. Echovirus 11 (E11) has been recently associated with more severe clinical features, including inflammatory diseases, acute flaccid paralysis (AFP), severe acute hepatitis, aseptic meningitis, and coagulation disorders [5,6,7]. The European Center for Disease Prevention and Control (ECDC), in conjunction with the World Health Organization (WHO), has observed a growing incidence of severe infections in newborns during 2022–2023 linked to the new E11 lineage 1, which emerged from recombination events that occurred in 2022 [8]. The new E11 variant has been detected in France, Croatia, Italy, Spain, Sweden, and in the United Kingdom of Great Britain and Northern Ireland [8]. According to the ECDC, E11 infection should be factored into the differential diagnosis for conditions like hepatitis, sepsis, myocarditis or pericarditis, and critical neurological and respiratory illnesses in infants, to accurately assess its public health implications (Item ID 2023-EIP-00026) [9].

Currently, there are no targeted pharmaceutical therapies approved for infections caused by non-polio EV, and creating a vaccine that covers all EV serotypes is not achievable due to the high antigenic variability [5]. The availability of several anti-EV compounds targeting viral or cellular factors has been demonstrated by in vitro studies, including enviroxime, which prevents the synthesis of positive-strand RNA by blocking the assembly of the replication complex [10]. Despite the latest progress, most of the research is focused on discovering compounds active against *Enterovirus* 71 (EV-71) and *Enterovirus* 30 (EV-30), which are often associated with meningitis and coagulation disorders [11]. Therefore, the increasing need for new effective compounds with anti-EV activity is imperative [12]. This study seeks to uncover new compounds that may exhibit antiviral properties while also enhancing our comprehension of the mechanisms that drive their effectiveness against EV viruses.

An attractive target for the discovery of novel antiviral drugs is represented by the RNA-dependent RNA polymerase (RdRp) enzyme, which plays a crucial role in the replication cycle of most RNA viruses [13]. This enzyme is highly conserved among evolutionary distant RNA viruses and does not have a homolog in mammalian cells. These characteristics, together with the extensive knowledge of RdRp structure and functions, and the easy availability of biochemical assays for the rapid screening of large libraries of compounds, make this enzyme an optimum target for new antiviral molecules.

In a previous study, Leusciatti et al. [14] reported the anti-SARS-CoV-2 RdRp activity of newly synthesized isoxazoline-carbocyclic monophosphate nucleotides, determined through computational analysis together with RdRp inhibition and cytotoxicity biological assays. The results obtained showed that the examined compounds could bind the SARS-CoV-2 RdRp nucleotide binding pocket, significantly reducing RNA synthesis [14]. This study aimed to investigate the potential anti-E11 activity of two compounds, **4a** and **4b** (isoxazoline-carbocyclic monophosphate nucleotides), previously shown to exhibit moderate anti-SARS-CoV-2 RdRp activity [14], using in vitro biological assays.

The synthetic approach was based on pericyclic reactions, including hetero Diels–Alder cycloaddition of nitrosocarbonyl intermediates and 1,3-dipolar cycloaddition of nitrile oxides. These strategies enabled the derivatization of aromatic substituents, construction of specific heterobases (e.g., 6-chloropurine), and the selection of the most suitable phosphorylation method to obtain the final nucleotide analogs **4a** and **4b** [15].

## 2. Materials and Methods

### 2.1. Tested Compounds

Two isoxazoline-carbocyclic monophosphate nucleotides (**4a** and **4b**) were designed and synthesized through the HDA cycloaddition of suitably prepared nitrosocarbonyl intermediates and cyclopentadiene, followed by the 1,3-dipolar cycloaddition reaction of the obtained dipolarophile with the stable anthracenenitrile oxide. The derivatization of the antracene ring with bromine conducted on the regioisomeric cycloadducts and the linear construction of the 6-chloropurine rings will afford the desired nucleosides. These are the precursors of the nucleotides of type **4a**, **b** that can be obtained through phosphorylation according to properly chosen protocols. Compounds **4a** and **4b** were solubilized in dimethyl sulfoxide (DMSO), stored at −20 °C, and the final dilutions for the experiments were carried out in Dulbecco’s modified Eagle’s medium (DMEM; Sigma-Aldrich, St. Louis, MO, USA).

### 2.2. Cells and Viruses

Epithelial monkey kidney VERO 76 cells [ATCC CRL 1587, *Cercopithecus aethiops*] were maintained in DMEM supplemented with 1% penicillin-streptomycin, and 2 mM L-glutamine (Sigma-Aldrich), and 10% fetal bovine serum (FBS; Sigma-Aldrich), at 37 °C and 5% CO_2_. Echovirus 11 lineage 1 [16] was maintained in our laboratory and propagated in VERO 76 cells. The virus was stored in small aliquots at −80 °C until use. All experimental work involving viruses was performed in an appropriate biosafety level containment laboratory.

### 2.3. Cytotoxicity Assays

According to the manufacturer’s instructions, cell viability was measured using the MTS ((3-(4,5-dimethylthiazol-2-yl)-5-(3-carboxymethoxyphenyl)-2-(4-sulfophenyl)-2H-tetrazolium) tetrazolium assay (CellTiter 96^®^ AQueous One Solution, Promega, Madison, WI, USA). Briefly, VERO 76 cells were seeded in 96-well plates at an initial density of 2 × 10^4^ cells/mL in complete medium. After overnight incubation at 37 °C/5% CO_2_, cells were treated with base-2 serial dilutions (0.781–100 µM) of compounds **4a** and **4b**, prepared from 10 mM stock solutions in DMSO. For each concentration, a vehicle-only control containing the same final DMSO percentage (up to 1% *v/v* at 100 µM) was included. After 48 h of incubation, 20 µL of MTS reagent were added directly to each well and the plates were incubated according to the manufacturer’s protocol. The conversion of MTS into a soluble formazan product by metabolically active cells was quantified by measuring the absorbance at 490 nm. For data analysis, the optical density (OD) of each well was first corrected by subtracting the background OD (medium + MTS without cells). The percentage of cell viability was then calculated by comparing the corrected OD of treated samples with the mean corrected OD of untreated control cells (0 µM), which was set as 100% viability:

Cell viability (%) = (OD_treated − OD_blank)/(mean OD_control − OD_blank) × 100

Two independent experiments were performed, each in triplicate (*n* = 6 per condition). Dose–response curves were generated and CC_50_ values were determined by nonlinear regression using GraphPad Prism software v. 8.0 (San Diego, CA, USA).

In addition to the MTS assay, cytotoxicity of the compounds under study has also been assayed by optical microscopy analysis using the same method described below for the CPE inhibition assay.

### 2.4. Cytopathic Effect Inhibition Assay

Protection of VERO 76 cells from E11-induced cytopathic effect (CPE) was evaluated using a CPE inhibition assay at a multiplicity of infection (MOI) of 0.025. In this assay, increased crystal violet staining indicates preservation of cell viability, reflecting protection against virus-induced cytopathic damage mediated by the tested compounds. The MOI of 0.025 was selected to allow multi-cycle infection, thereby maximizing the detection of compounds that limit viral propagation. Under these conditions, complete CPE was consistently observed in VERO 76 cells within 48 hours.

(A) A co-treatment assay was performed as a screening test to determine the antiviral activity of **4a** and **4b** as anti-E11 agents. Each compound was added to the cell monolayer (3.2–100 μM) at the same time as viral infection for 48 h at 37 °C.
(B) A post-infection assay was performed to assess the possible ability of **4a** and **4b** to interfere with the viral replication due to the RdPR binding. VERO 76 cells in 96-well plates were infected with E11 for 1 h in DMEM at 37 °C to allow viral adsorption and penetration. After removal of the virus inoculum, the cells were washed once with culture medium and treated for 48 h with different concentrations of **4a** and **4b** (3–100 μM).

Each treatment was performed in triplicate. For all assays, VERO 76 cells were seeded in 96-well plates at a density of 2 × 10^4^ cells/well and allowed to form confluent monolayers by overnight incubation at 37 °C. After 48 h, CPE inhibition was evaluated by optical microscopy observation. Cells were fixed with 4% formaldehyde for 15 min at room temperature and stained with 0.1% (*w*/*v*) crystal violet for 30 min. The absorbance was measured at 595 nm.

Dose–response curves derived from these absorbance values were used to determine the half-maximal effective concentration (EC_50_) for each compound. Although no antiviral drugs are approved for enterovirus infections, Remdesivir (Cat. No. HY-104077, MedChemExpress, Shanghai, China) was included for comparison based on its documented in vitro activity against other enteroviruses and its mechanism of action targeting the viral RNA-dependent RNA polymerase. All experiments were carried out in triplicate.

### 2.5. Plaque Assay

E11 infectious titers were determined by plaque assay. Briefly, 100 µL of culture supernatant from infected cells were serially diluted (10-1–10-4) in DMEM containing 1% FBS. VERO 76 cells were seeded in 24-well plates 24 h before infection to obtain 90% confluency. Cells were inoculated with 50 µl of each dilution and incubated for 1 h at 37 °C with gentle rocking. After adsorption, the inoculum was removed, and cells were overlaid with DMEM containing 1% FBS and carboxymethyl cellulose (CMC). Following 72 h incubation, monolayers were fixed, stained with crystal violet, and plaques were counted. Viral titers were expressed as plaque-forming units per milliliter (PFU/mL) according to:

PFU/mL = (number of plaques)/(dilution factor × inoculated volume).

### 2.6. E11 Genome Quantification in Infected VERO 76 Cells and Supernatant

Quantification of E11 genomic RNA was used as a hallmark of RNA virus replication within VERO 76-infected cells. The viral RNA was extracted from both cell culture supernatants and cell-associated fractions and quantified by one-step qRT-PCR using the automated Elite InGenius one-step RNA Enterovirus ELITe MGB^®^ Kit, according to the manufacturer’s protocols. The experiment was performed in triplicate and RNA yield was reported as the mean value of a triplicate assay. To improve the accuracy of extracellular RNA quantification, particularly at 48 h post-infection (p.i.), a secondary analysis was performed using a two-step SYBR Green RT-qPCR. In particular, specific primers targeting the gene encoding the RNA-dependent RNA polymerase (RdRp) of E11 were designed using the Primer3 software (version 4.1.0). The sequences were as follows: Forward primer: 5′-TGCAAGGAAAAGGGGTGGTT-3′ and Reverse primer: 5′-AGAGACAAAGGTGGTGAGCG. To account for variability in RNA recovery and cDNA synthesis, an in-house transcribed synthetic RNA was added to each sample prior to RNA extraction [17]. Reverse transcription was carried out with a high-capacity cDNA synthesis kit, followed by quantitative PCR using SYBR Green Master Mix on a real-time PCR instrument. Data were analyzed using the ΔΔCt method, with normalization to the synthetic RNA spike-in.

### 2.7. Molecular Docking Studies

The chemical structures of compounds **4a** and **4b** were drawn using ChemDraw (freeware version 12.01, PerkinElmer Waltham, MA, USA) and converted into PDB format. These structures were refined, and all water molecules and non-essential ligands were removed using AutoDockTools (Scripps Research Institute, MGLTools, version 1.5.7; La Jolla, CA, USA). Since no crystal structure of E11 RdRp was available, a homology model was built using the Swiss-Model server, an automated platform for comparative 3D protein modeling. The model was based on the RNA polymerase of Coxsackievirus B3 (PDB ID: 4Y2A), which showed the highest sequence identity and resolution among the available templates.

Molecular docking studies were performed using the AutoDockTools 1.5.7 software (Scripps Research Institute, MGLTools, version 1.5.7; La Jolla, CA, USA), with which initially all small molecules, such as water, salts, and ligands, were removed. The docking protocol using AutoDockTools (Scripps Research Institute, MGLTools, version 1.5.7; La Jolla, CA, USA) was constructed using a box with dimensions of 15 × 15 × 15 Å, centered on coordinates [60.8, 44.7, 58.8] with a grid spacing of 1 Å, using the default settings of the Lamarckian genetic algorithm. To best center the grid box, the coordinates of the complex ligand were extrapolated from the crystallographic structure of 4Y2A and were used for our analysis. The protein–ligand interactions were analyzed and visualized using the Pymol (version 2.6, Schrödinger, Portland, OR) and BIOVIA Discovery Studio 2024 ( version 24.1.0.23298, Dassault Systèmes, San Diego, CA, USA) software.

### 2.8. Statistics

All statistical analyses were performed using GraphPad Prism V.8.0 for Windows (GraphPad Software, San Diego, CA, USA). Data are presented as mean ± standard deviation (SD) from at least three independent experiments. Dose–response curves were generated to determine the CC_50_ values by nonlinear regression. Comparisons between groups were carried out using one-way ANOVA, followed by Bonferroni’s post hoc test for multiple comparisons. Differences were considered statistically significant at *p* < 0.05.

## 3. Results

### 3.1. Evaluation of Cytotoxicity of ***4a*** and ***4b*** in VERO 76 Cells

**Figure 1:**
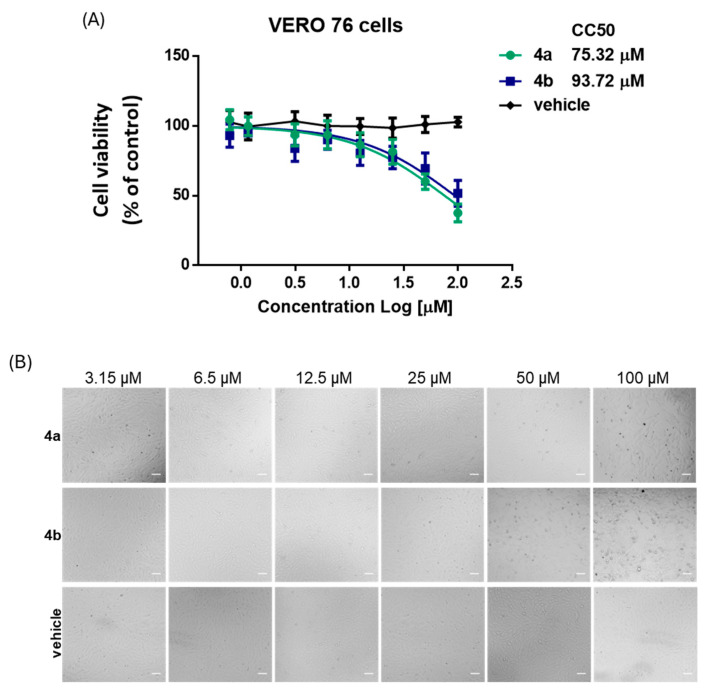
Evaluation of cytotoxic effects of nucleotides **4a** and **4b** on VERO 76 cells. (**A**) Cell viability was measured by MTS assay after 48 h treatment with base-2 serial dilutions (0.781–100 µM) of compounds **4a** and **4b**. Vehicle-only (DMSO) controls, matched to the solvent concentration present at each dose, were included. Results are expressed as percentage of viable cells relative to untreated controls (mean ± SD, *n* = 6). Nonlinear regression analysis yielded CC_50_ values of 75.3 ± 10.6 µM for **4a** and 93.7 ± 19.2 µM for **4b**. (**B**) Representative bright-field microscopy images of VERO 76 cells exposed for 48 h to increasing concentrations of **4a**, **4b**, or vehicle. Scale bar: 100 µm.

The potential cytotoxic effects of nucleotides **4a** and **4b** on VERO 76 cells were evaluated using the MTS tetrazolium assay and by microscopy observation. Cell metabolic activity was determined by the enzymatic reduction of MTS into a soluble formazan product. A dose–response assay using base-2 serial dilutions in the range 0.781–100 µM was performed to calculate the cytotoxic concentration that inhibited 50% of cell growth (CC_50_). Results showed that compounds **4a** and **4b** reduced VERO 76 cell viability in a dose-dependent manner, with CC_50_ values of 75.3 ± 10.6 µM and 93.7 ± 19.2 µM, respectively (Figure 1A). Compound **4a** maintained >80% viability up to 25 µM, whereas compound **4b** already displayed a modest reduction (~77%) at this concentration. Both compounds induced a marked decrease in viability at ≥50 µM (Figure 1B). Microscopic examination corroborated these results, revealing no appreciable morphological alterations in vehicle-treated cells or in those exposed to compounds at ≤25 µM, whereas cytotoxic changes became evident only at higher concentrations (≥50 µM)

### 3.2. Analysis of Compound-Mediated Protection from E11-Induced Cytopathic Effect

**Figure 2:**
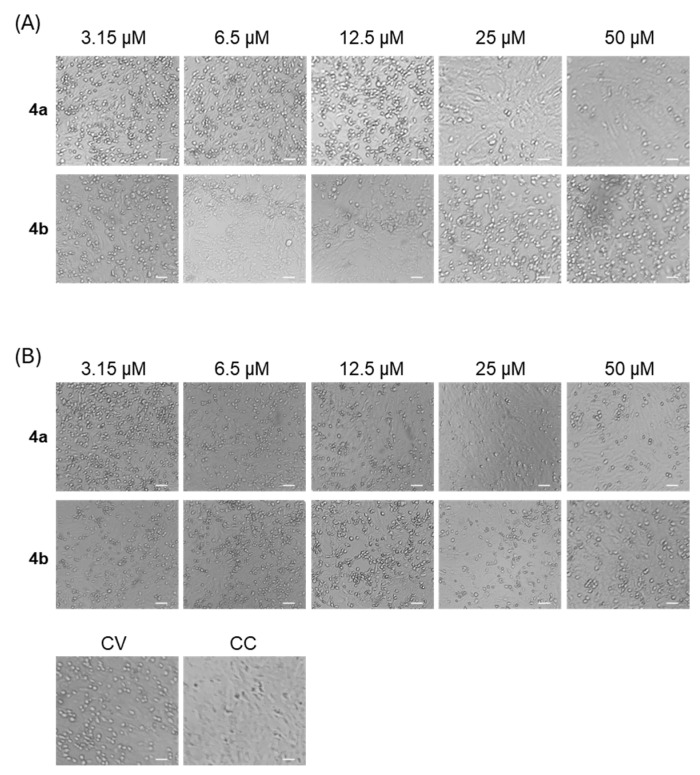
Morphology image of VERO 76 cells treated with compounds **4a** and **4b** at different concentrations (3.15–50 μM) in cotreatment (**A**) and post-infection (**B**) assays. Infected (CV) and untreated (CC) cells were used as positive and negative controls, respectively. Scale bar: 100 µm.

The ability of nucleotides **4a** and **4b** to protect VERO 76 cells against E11 infection was assessed through CPE reduction in co-treatment and post-infection antiviral screening assays at different concentrations ranging from 3.15 to 50 μM. The CPE reduction assay provides insight into the capacity of various compounds to hinder viral replication and infection, ultimately increasing cell viability. Microscopic observation revealed a decrement in E11 infection in both co-treatment and post-infection assays with a higher significant reduction observed in the post-infection assay (Figure 2).

**Figure 3:**
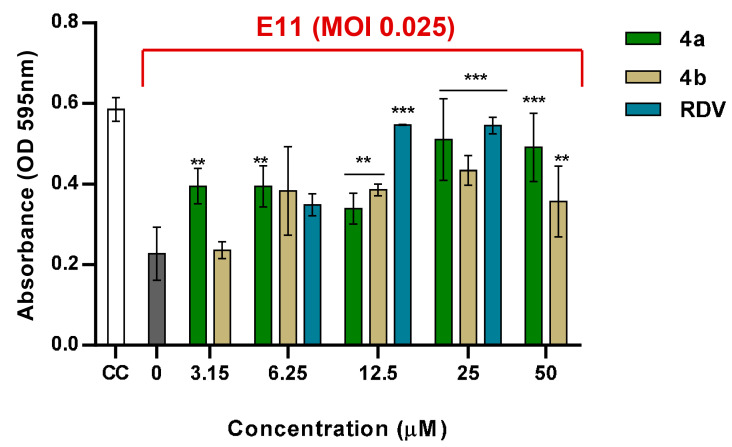
CPE inhibition assay showing protection of VERO 76 cells from E11-induced cytopathic effect by compounds **4a**, **4b** and RDV. VERO 76 cells were treated with **4a**, **4b** and RDV after viral infection (MOI 0.025). Mock-infected cells (CC, white bar) and infected untreated (0) cells served as negative and positive CPE controls, respectively. RDV was experimentally tested at three concentrations (from 6.25 to 25 µM) under the same conditions of compounds **4a** and **4b**. Data are expressed as mean ± SD of three independent experiments. Asterisks indicate statistically significant differences between treated samples (**4a**, **4b**, and RDV) and the infected untreated control (E11, 0 µM): ** *p* < 0.01, *** *p* < 0.001.

Morphologically, E11-infected VERO 76 cells showed a rounded appearance, and cells detached from the dish, whereas treatment with nucleotides **4a** and **4b** successfully diminished the CPE caused by the virus. Cell viability was evaluated by crystal violet staining. Among the tested conditions, the post-infection assay revealed the strongest antiviral activity for both compounds against E11. Therefore, this protocol was selected for subsequent experiments. Treatment with compound **4a** at 25 and 50 µM resulted in a mean reduction in E11 infection of 83.9% and 79.8%, respectively. In comparison, **4b** treatment at the same concentrations reduced E11-induced CPE by 58% and 38% (Figure 3). In the experimental setting, due to the absence of any known E11-specific antiviral compound to serve as a positive control, Remdesivir (RDV) was included for comparison, given its broad-spectrum activity against RNA viruses [18]. As shown in Figure 3, RDV was tested at three concentrations (from 6.25 to 25 µM), actually producing a robust and dose-dependent protection from E11-induced CPE, confirming assay reliability and allowing a direct comparison with the activity of compounds **4a** and **4b.**

To quantitatively evaluate the activity of compounds **4a** and **4b**, dose–response curves from the post-infection CPE inhibition assay were used to determine their half-maximal effective concentrations (EC_50_). Compound **4a** showed higher potency, with an EC_50_ of 8.15 ± 1.4 µM, whereas compound **4b** exhibited a weaker effect, with an EC_50_ of 40.5 ± 1.8 µM. Using these EC_50_ values together with the CC_50_ values obtained by MTS assay (73.32 µM for 4a and 93.72 µM for **4b**), the Selectivity Index (SI = CC_50_/EC_50_) was calculated. Compound **4a** displayed an SI of 8.99, while compound **4b** showed an SI of 2.31. Altogether, the results indicated that nucleotide **4a** is more effective in preventing E11 infection in VERO 76 cells than **4b**, supporting its selection for further evaluation.

### 3.3. Molecular Evaluation of Antiviral Activity on E11 Virus Infection

**Figure 4:**
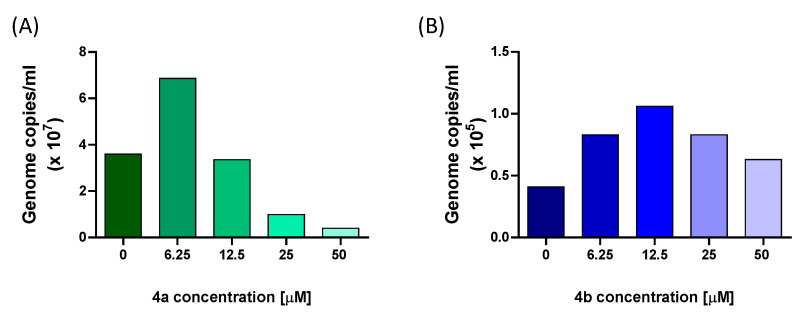
Quantification of viral RNA detected in the supernatant of VERO 76 cells infected with E11 after treatment with nucleotides **4a** and **4b**. VERO 76 cells infected with E11 at a multiplicity of infection (MOI) of 0.025 were incubated for 48 h in the absence (0) or presence of different concentrations of **4a** (**A**) and **4b** (**B**).

The antiviral activity of nucleotides **4a** and **4b** against E11 replication was further evaluated by quantifying viral RNA in the supernatant of infected VERO 76 cells treated with increasing concentrations of each compound (6.25–50 μM for **4a**; 6.25–100 μM for **4b**). Following viral infection at a MOI of 0.025, cells were treated and incubated for 48 h. Viral genome copies were then assessed by qRT-PCR. Treatment with nucleotide **4a** led to a dose-dependent reduction in extracellular viral RNA, with a 72% decrease observed at 25 μM and an 89% reduction at 50 μM relative to untreated infected controls. Conversely, **4b** did not markedly affect viral RNA levels at any of the tested concentrations (Figure 4). These findings are consistent with the observed decrease in CPE and confirm the superior antiviral activity of compound **4a.**

### 3.4. Time-Course Analysis of Viral Replication Inhibition by ***4a***

Given the consistent and marked antiviral activity exhibited by **4a**, a time-course analysis was performed to further characterize its effects on E11 replication dynamics. To evaluate the antiviral activity of compound **4a** in VERO 76 cells over time, viral replication was assessed through quantification of viral genome copies via qRT-PCR in both the cell-associated and supernatant fractions, as well as by CPE analysis.

**Figure 5:**
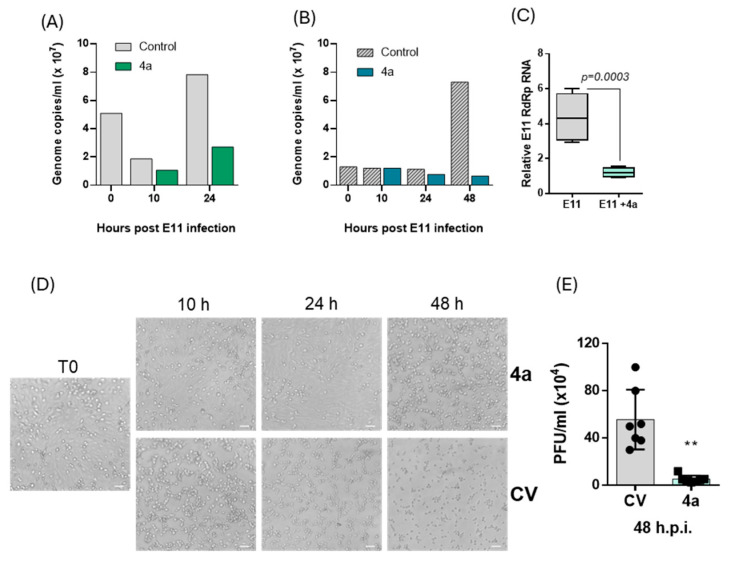
Time-course analysis of **4a**-mediated inhibition of E11 replication. VERO 76 cells infected with E11 at a MOI of 0.025 were incubated in the absence (control samples) or presence of **4a** at 25 μM and harvested at the indicated time points p.i. Total RNA was extracted and quantified from monolayer cells (**A**) and culture supernatants (**B**). Data shown in panels **A** and **B** refer to cumulative RNA extracted from pooled samples of three independent experiments. (**C**) RT-qPCR analysis of viral RNA (targeting the gene encoding the RdRp)in the culture supernatant at 48 h p.i., performed on three independent biological replicates. Data are shown as mean ± SD calculated by one-way ANOVA and Bonferroni’s post hoc test. (**D**) Assessment of viral infectivity by monitoring cytopathic effect (CPE). Supernatants collected at 10, 24, and 48 h p.i. from control (CV) or **4a**-treated infected cultures were used to infect fresh VERO 76 cells. Phase-contrast microscopy images were captured at 48 h p.i. T0 represents uninfected cells. Scale bar: 100 µm. (**E**) Infectious viral titer (PFU/mL) measured by plaque assay on supernatants collected at 48 h p.i. from untreated (CV) and 4a-treated cultures (** *p* < 0.01).

At different points post-infection, E11 RNA levels were measured within the cell monolayer (Figure 5, panel A) and in the culture supernatant (Figure 5, panel B), which reflects newly produced and released viral particles. Overall, qRT-PCR analysis of intracellular RNA extracted from E11-infected untreated VERO 76 cells revealed an initial decline in viral load between 0 and 10 h post-infection (p.i.), followed by a marked increase in intracellular E11 RNA from 10 to 24 h p.i.

In parallel, a marked increase in E11 RNA levels was observed in the culture supernatant at 48 p.i., consistent with active viral replication and extracellular release of viral particles.

Based on previous findings suggesting 25 μM as the most effective concentration for achieving maximum infection protection with reduced cytotoxicity, this specific concentration was chosen to investigate the antiviral efficacy of **4a**. The amount of viral RNA measured in the intracellular and the supernatant fractions was compared against the control group, which consisted of VERO 76 cells that were infected but not subjected to **4a** treatment. The treated group showed a significant drop in intracellular viral RNA levels, with reductions of 43% at 10 h and 65% at 24 h p.i. (Figure 5A). The inhibition rate observed in the supernatant was consistent with the intracellular findings, with a 31% drop in viral RNA levels after 24 h and a remarkable 91% decline at 48 h p.i. (Figure 5B).

To further corroborate and confirm the inhibitory effect of **4a** on viral RNA release into the extracellular environment, an additional quantification was performed using a two-step SYBR Green-based RT-qPCR targeting the gene ecoding for the viral RdRp. RNA was extracted from culture supernatants collected at 48 h p.i., and a synthetic RNA spike-in was included to normalize for extraction and reverse transcription variability [19]. As shown in Figure 5C, RdRp RNA levels in the supernatant of **4a**-treated cells were significantly reduced compared to untreated controls (*p* = 0.0003), reinforcing the evidence of a potent antiviral effect on extracellular viral genome production.

To evaluate the infectivity of the produced viral progeny, supernatants from **4a**-treated and untreated cultures were used to infect fresh VERO 76 cells. As shown in Figure 5D, cells exposed to supernatants from **4a**-treated cultures displayed a markedly reduced CPE compared to those infected with supernatants from untreated controls, indicating a substantial loss of infectious particles. To directly quantify this effect, a plaque assay was performed on supernatants collected at 48 h p.i. As shown in Figure 5E, the infectious viral titer (PFU/mL) was dramatically reduced in the presence of **4a**, showing a >10-fold decrease compared to the untreated control (CV). These data confirm that the antiviral activity of **4a** not only limits extracellular viral RNA but also profoundly impairs the production of infectious E11 progeny.

### 3.5. In Silico Binding Affinity Analyses of ***4a*** and ***4b*** to RdRp

To complement our biological findings and gain insights into the mechanisms underlying the inhibitory activity of **4a** against E11 replication, we generated a homology-based 3D model of the E11 RdRp and performed in silico molecular docking to predict the binding poses of compounds **4a** and **4b** within the polymerase catalytic site.

The chemical structures of compounds **4a** and **4b** were initially generated using ChemDraw (freeware version 12.01, PerkinElmer Waltham, MA, USA) (Supplementary Figure S1). Since no crystallographic structure of E11 RdRp was available in public databases, a homology model was generated using the Swiss-Model online platform. The amino acid sequence of the E11 RNA-dependent RNA polymerase was used as input, and 50 template structures were identified. Among them, the RdRp of Coxsackievirus B3 (PDB ID: 4Y2A) was selected based on the highest identity score and optimal resolution in angstroms. The resulting 3D structure was used for molecular docking studies (Figure S2).

**Figure 6:**
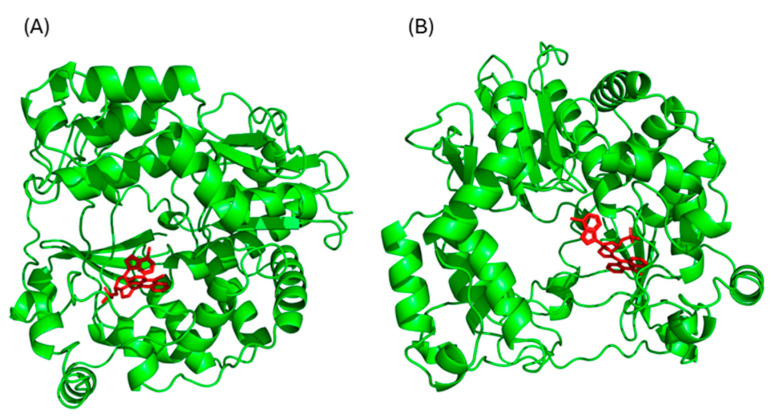
Three-dimensional structure, obtained with Pymol software (version 2.6, Schrödinger, Portland, OR, USA), of **4a** molecule (**A**) and **4b** molecule (**B**) docked into RNA-dependent RNA polymerase of E11.

The most probable 3D binding poses predicted for compounds **4a** and **4b** are shown in Figure 6. Detailed docking scores and binding energy distributions, including cluster populations for each conformation, are provided in Supplementary Figure S3.

The higher molecule affinity of **4a** to E11 RpRd, compared to that of **4b**, could be related to most energetically favorable conformation corresponding to the lowest binding energy (−8.89 Kcal/mol vs. −8.11 Kcal/mol) (Figure S3A,B). This data was also supported by the AutodockTools analyses, indicative of inhibitor ligand concentration which occupies the 50% of the receptor sites in absence of competitors, which showed inhibition constant values of 302.19 nM for the **4a** molecule and 1.14 µM for the **4b** molecule (Figure S3A,B).

**Figure 7:**
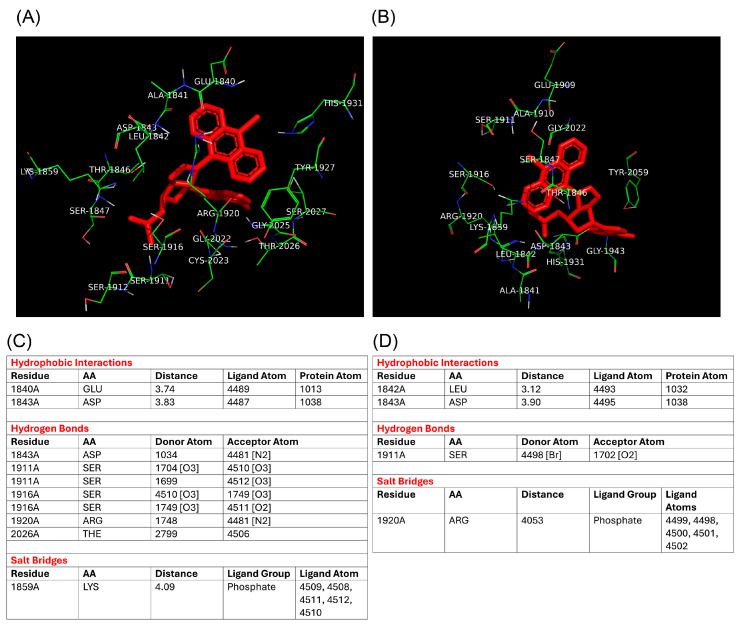
Binding interactions of compounds **4a** and **4b** with E11 RdRp. (**A**,**B**) Predicted 3D binding modes of compounds **4a** (**A**) and **4b** (**B**) within the E11 RdRp active site, visualized in PyMOL (version 2.6, Schrödinger, Portland, OR, USA). (**C**,**D**) Summary of molecular interactions between **4a** (**C**) and **4b** (**D**) and E11 RdRp.

Moreover, Figure 7 showed amino acid interactions with Arg1920 and Ser1911 via conventional hydrogen bonds involving the oxygen and nitrogen atoms of compound **4a** (Figure 7A,C), and with Arg1920 through hydrogen bond with oxygen of the phosphate group of the **4b** molecule or a halogen bond of Ser1911 with the Bromine of the **4b** molecule (Figure 7B,D). A 2D schematic representation of the molecular interactions between compound **4a** and E11 RdRp is shown in Supplementary Figure S4.

To conclude, this retrospective docking analysis of the interaction between the **4a** and **4b** molecules with RdRp of E11 showed a higher interaction of the **4a** molecule with respect to **4b**, strongly supporting the inhibition data obtained in the biological assay, as well as confirming the RdRp enzyme as the molecular target for the assayed compounds.

## 4. Discussion

Enteroviruses continue to significantly impact global health, particularly related to the emergence of novel E11 variants [5,7]. This alarming development has spurred increased efforts toward repurposing existing drugs and developing novel antiviral therapies targeting enteroviruses [20,21,22]. Although numerous compounds have demonstrated promising anti-enteroviral activity in vitro, only a limited number have shown efficacy in vivo [12,13,14,23,24]. Among those, only few viral capsid inhibitors (disoxaril, pleconaril, pirodavir, vapendavir and pocapavir) have progressed to clinical evaluation [25,26,27,28]. However, their therapeutic use is frequently limited by adverse effects such as crystalluria, menstrual irregularities, and the emergence of drug-resistant viral variants [25,28]. Thus, we are still far from having identified compounds capable of providing an optimal therapeutic treatment for enterovirus infections, including those caused by echovirus variants. Non-structural proteins represent innovative targets for the development of broad-spectrum antivirals. Notably, recent studies have shown that both coronaviruses and enteroviruses share a conserved mechanism for initiating viral RNA synthesis, which involves the covalent modification of specific non-structural proteins (nsps) [29]. In particular, in echoviruses, the viral protein genome-linked (VPg) protein can initiate the RNA synthesis after its cleavage by the viral protease 3 CD and covalent attachment of uridine monophosphate (UMPylation) by 3D^pol^ [30]. Similarly, the non-structural protein 12 of coronaviruses (CoV RdRp nsp12) uridylates the non-structural protein 8 (nsp8), which then primes RNA synthesis from a poly(A) template.

A class of promising antiviral drugs under investigation is represented by molecules inhibiting RdRp complex formation through binding with specific nsps 28.

In general, the interaction between antiviral compounds and viral non-structural proteins results in the inhibition of key enzymatic functions essential for the viral replication cycle. Within this class of antivirals are the two isoxazoline-carbocyclic monophosphate nucleotides, **4a** and **4b**, which were originally designed and synthesized as inhibitors of non-structural protein 12 (nsp12) of SARS-CoV-2 [11]. These compounds were shown to bind putatively to allosteric sites on nsp12 distinct from those targeted by Remdesivir, leading to a measurable reduction in RdRp activity. These findings support the potential of **4a** and **4b** as novel antiviral agents, warranting investigation of their efficacy against other RNA viruses, such as enteroviruses.

Given the clinical relevance of the new E11 variant and the conserved RNA synthesis mechanism, in this study, the potential anti-E11 activity of nucleotides **4a** and **4b** was tested by in vitro infection. In line with the results previously observed on Hep-2 and HepG2 cell lines, exposure of cells to high concentrations of **4a** and **4b** is associated with a certain cytotoxicity. 

According to a previous study investigating the inhibition of Echovirus 71 RdRp by the adenosine analog NITD008 [31], the ability of nucleotides **4a** and **4b** to interfere with E11 viral replication was further assessed by measuring the levels of viral RNA in the supernatant by qRT-PCR. The results showed that incubation with **4a** caused a reduction in viral load by 72% at 25 μM and 89% at 50 μM. Differently, treatment with nucleotide **4b** resulted in a weaker inhibition of E11 infection. These findings confirmed, at the molecular level, the observations regarding the reduction in CPE and affirmed the more potent inhibitory properties of nucleotide **4a** against E11. In line with these observations, quantitative analysis of dose–response curves showed that **4a** displayed a markedly lower EC_50_ and a more favorable Selectivity Index compared to **4b**, further supporting the superior antiviral profile of **4a**. Due to the limited information on the E11 life cycle, a quantification of the viral RNA was conducted at different time points from T0 to 48 h p.i. The E11 RNA load was analyzed in the VERO 76 cells supernatant, which mainly contained released infectious particles. In line with Echovirus 71 and Echovirus 30 in vitro infection studies [3], and in accordance with the CPE assay, the results of this experiment revealed an initial decrease in viral load in the supernatant from T0 to 10 h p.i., followed by an increase in E11 RNA from 10 h to 48 h p.i. Since we hypothesized that compound **4a** could act by inhibiting the RNA-dependent RNA polymerase (RdRp), which is translated and becomes active in enteroviruses no earlier than 3–4 h post-infection, in this study we focused our kinetic analysis on timepoints from 10 h onwards. Earlier phases, such as viral adsorption, uncoating, and initial translation of the viral polyprotein, occur within the first 1–2 h p.i. and are not directly targeted by RdRp inhibitors. Nevertheless, future experiments including intermediate timepoints such as 6 h p.i. may help to define the onset of antiviral activity with greater temporal resolution.

The evaluation of viral progeny generation carried out in the presence of nucleotide **4a** at 25 μM showed a remarkable drop of E11 RNA load in supernatant of the treated group compared to the control at 24 h (31%) and 48 h p.i. (91%). According to the RNA load decrease in the supernatant, a reduction in intracellular E11 RNA was observed in the treated group. Altogether, the molecular analysis coupled with the CPE reduction assays results strongly suggest the inhibitory activity of nucleotide **4a** on both E11 RNA synthesis and viral particle release. To validate whether the reduction in viral RNA corresponded to a loss of infectious virus, we performed a plaque assay on supernatants collected at 48 h p.i. The marked decrease in PFU/mL in **4a**-treated cultures confirmed a profound impairment in the production of infectious E11 progeny, strengthening the antiviral evidence obtained through CPE and qRT-PCR analyses. Finally, the yield of E11 viral RNA in cells subjected to **4a** treatment was quantified over time. The results confirmed a continuous decrease in E11 genome levels in the supernatant while the quantification of genome inside the cell monolayer revealed a 60% decrease at 10 h p.i. followed by an E11 genome accumulation at 48 h. The increase in viral RNA observed within cells at 48 h could be explained as the result of the incomplete suppression of RNA synthesis by nucleotide **4a**, and the detection of genome fragments which will not become infectious virus particles.

In parallel with the antiviral evaluation, the cytotoxic potential of the compounds was assessed using the MTS assay. The analysis confirmed a dose-dependent reduction in cell viability, with CC_50_ values of 75.3 ± 10.6 µM for **4a** and 93.7 ± 19.2 µM for **4b**. Compound **4a** maintained a cell viability above 80% up to 25 µM, whereas for compound **4b** a modest reduction (~77%) was already observed at the same concentration. Microscopic inspection corroborated these data, showing no appreciable morphological alterations under these conditions. Overall, the pronounced antiviral effects of **4a** at sub-cytotoxic concentrations clearly outweighed the modest impact on cell viability, highlighting its favorable selectivity profile. By contrast, the narrower therapeutic window of **4b**, combined with its weaker antiviral activity, further supports **4a** as a more promising candidate for subsequent investigations.

Despite the lack of a crystallographic structure for E11 RdRp, a homology-based model using the closely related Coxsackievirus B3 RdRp provided a reliable structural framework for docking simulations. Compound **4a** exhibited a lower binding energy (−8.89 kcal/mol) compared to **4b** (−8.11 kcal/mol), and a stronger predicted inhibition constant (302.19 nM vs. 1.14 µM), suggesting a tighter interaction with the viral polymerase. Additionally, molecular docking identified specific interactions of **4a** with catalytically relevant residues such as Arg1920 and Ser1911, highlighting potential key contacts responsible for its inhibitory activity. These in silico observations are in line with the biological assays and support the hypothesis that RdRp is the molecular target of compound **4a** in E11.

In conclusion, the results of this study provided preliminary insights into the kinetics of infection of the new Echovirus 11 variant in VERO 76 cells. Moreover, the antiviral screening tests conducted in this study support the promising antiviral properties of the two monophosphate nucleotides **4a** and **4b** previously identified as possible SARS-CoV-2 inhibitors. In particular, the data obtained revealed the effectiveness of **4a** against additional RNA viruses, especially against the new Echovirus 11 variant.

Although additional investigations are required to broaden the insights on the mechanism of action of these molecules, our findings strongly indicate that nucleotide **4a** is an RdRp inhibitor, demonstrating its capability to cause dramatic reductions in E11 genome replication and viral particle formation in VERO 76 cells.

## Data Availability

The original contributions presented in the study are included in the article/Supplementary Material, further inquiries can be directed to the corresponding author.

## Supplementary Materials

The following supporting information can be downloaded at: https://www.mdpi.com/article/10.3390/microorganisms13122662/s1, Figure S1: Chemical structures of compounds **4a** (A) and **4b** (B); Figure S2: Chemical structure of the created RNA-dependent RNA polymerase of Echovirus11; Figure S3: Docking score energies and chemical parameters; Figure S4: 2D interactions of the **4a** molecule.
