# Supplementary material for: Unraveling the Potential Antiviral Activity of Isoxazoline-Carbocyclic Monophosphate Nucleotides Against Echovirus 11
Source: Microorganisms. 2025 Nov 23;13(12):2662. doi: 10.3390/microorganisms13122662 (PMC12734540; doi:10.3390/microorganisms13122662)
Supplement: Supplementary file 1 [file microorganisms-13-02662-s001.zip › microorganisms-3769354-supplementary.pdf]

SUPPLEMENTARY DATA

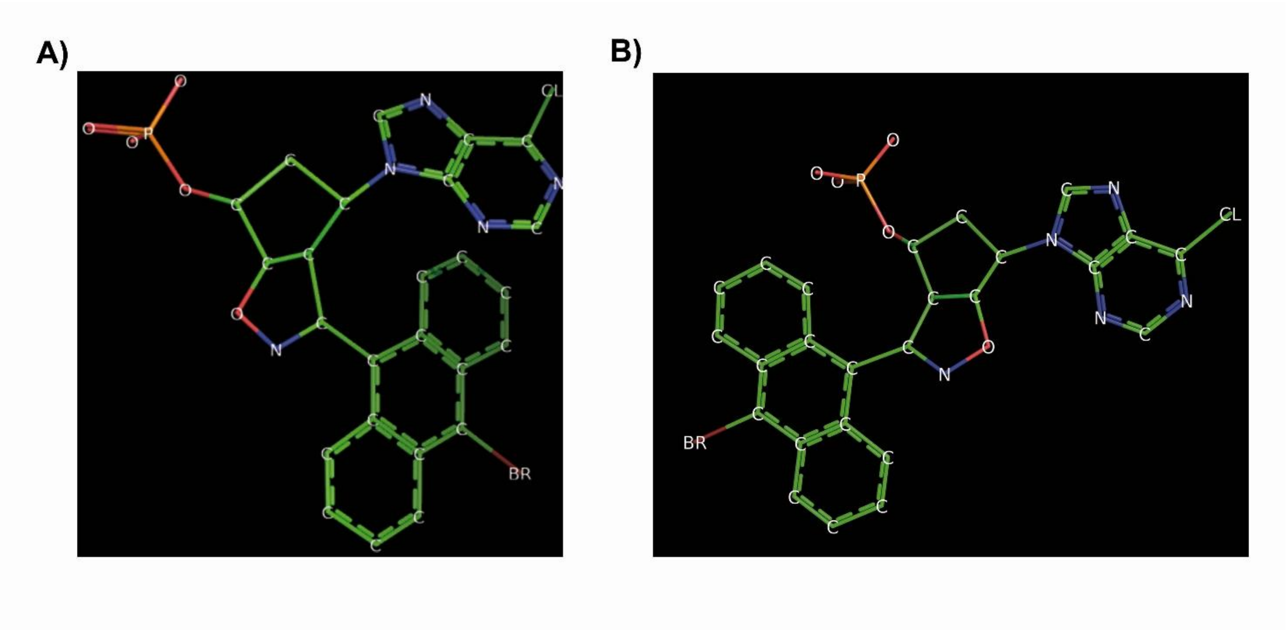

Figure S1. Chemical structures of ***4a*** *(A) and* ***4b (B)*** *compounds obtained with* ***ChemDraw and Pymol softwares.***

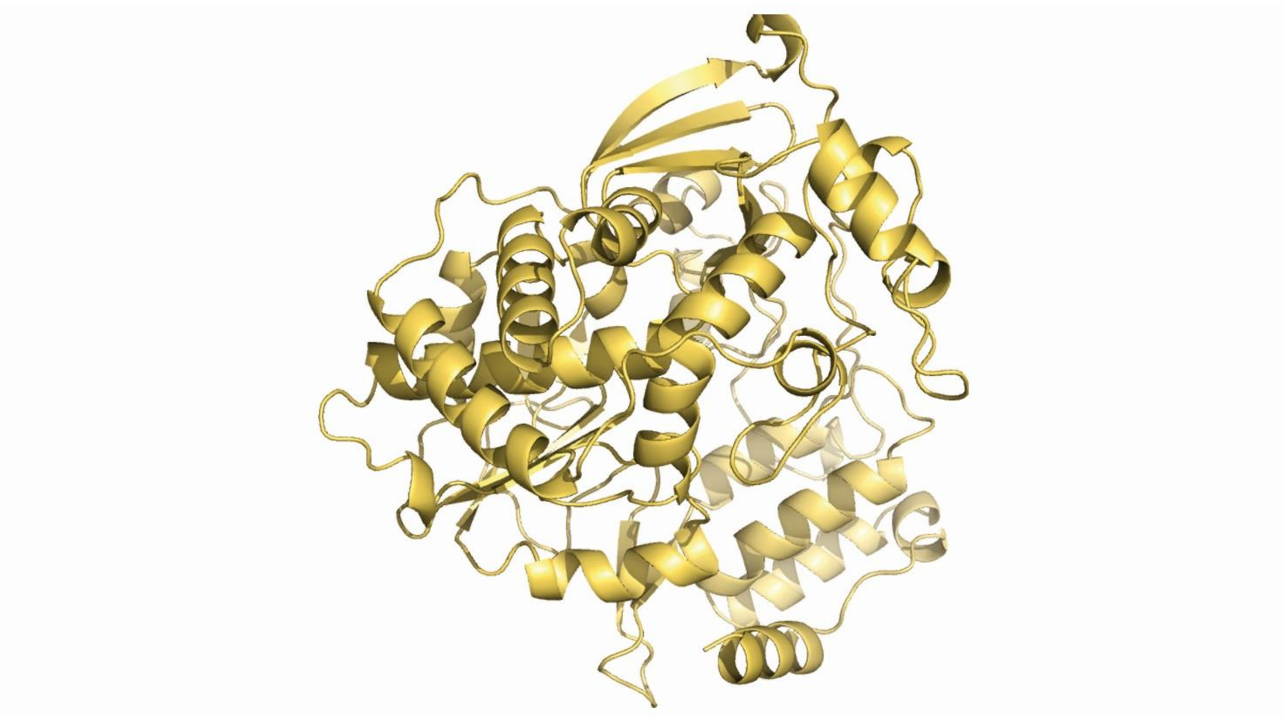

*Figure S2. Chemical structure of the created RNA-dependent RNA polymerase of Echovirus11 obtained with **Pymol software**.*

A)

Clus -ter Rank	Lowest Binding Energy	Run	Mean Binding Energy	Num in Clus
1	-8.89	10	-8.89	1
2	-8.43	8	-8.03	4
3	-8.31	5	-8.22	2
4	-8.03	1	-8.03	1
5	-7.12	7	-6.95	2

binding_energy=-8.89
ligand_efficiency=-0.23
inhib_constant=302.19
inhib_constant_units=nM
intermol_energy=-10.09
vdw_hb_desolv_energy=-7.4
electrostatic_energy=-2.69
total_internal=-2.02
torsional_energy=1.19
unbound_energy=-2.02
filename=1.dlg
clRMS=0.0
refRMS=90.76
rseed1=None
rseed2=None

B)

Clus -ter Rank	Lowest Binding Energy	Run	Mean Binding Energy	Num in Clus
1	-8.11	4	-8.11	1
2	-7.40	8	-7.16	6
3	-6.81	10	-6.81	1
4	-6.80	3	-6.79	2

binding_energy=-8.11
ligand_efficiency=-0.21
inhib_constant=1.14
inhib_constant_units=uM
intermol_energy=-9.3
vdw_hb_desolv_energy=-5.36
electrostatic_energy=-3.94
total_internal=-1.54
torsional_energy=1.19
unbound_energy=-1.54
filename=1.dlg
clRMS=0.0
refRMS=87.73
rseed1=None
rseed2=None

*Figure S3. Docking score energies and chemical parameters from the **AutodockTools software** for **4a** molecule (**A**) and **4b** molecule (**B**).*

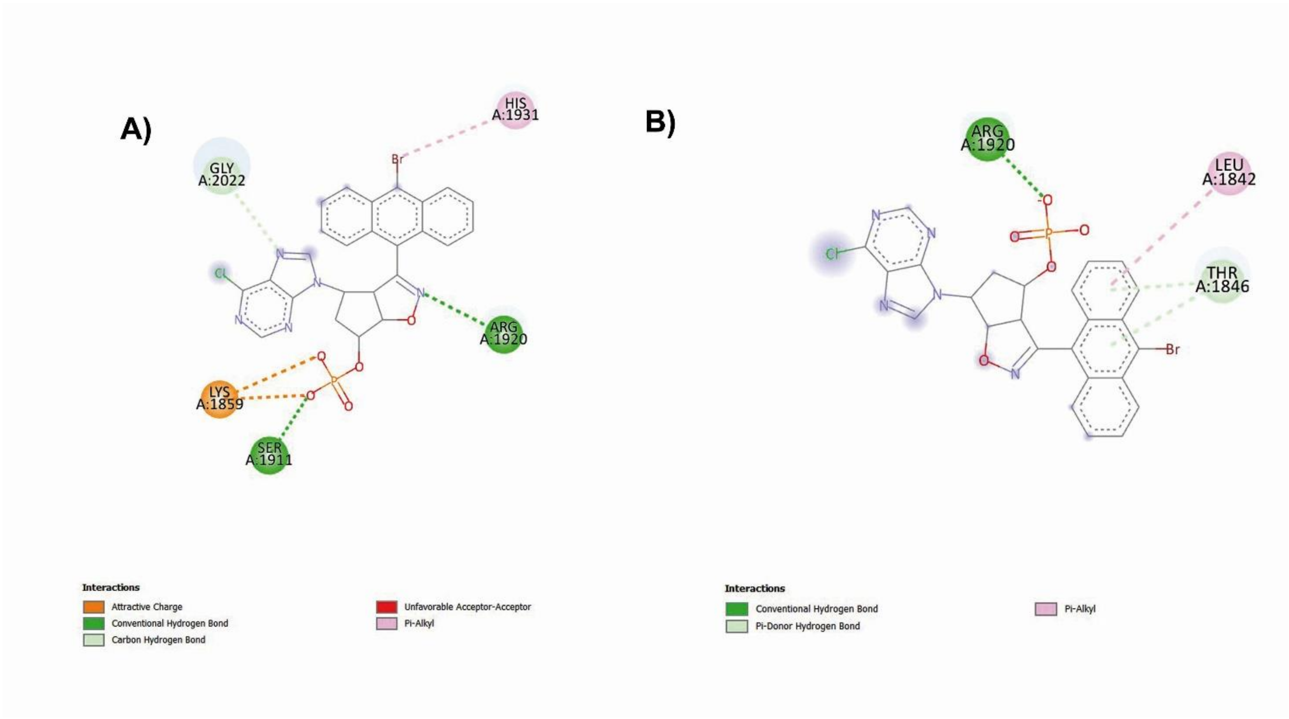

*Figure S4. 2D interactions of **4a** molecule with enzyme obtained from **Discovery Studio software**, with greater chemical interaction between **4a** molecule (**A**) or **4b** molecule (**B**) and Echovirus11 enzyme.*
